# Watch Me Play!: results of a feasibility study of a remotely delivered intervention to promote mental health resilience for children (age 0–8 years) across UK early years and children’s services

**DOI:** 10.1192/bjo.2026.12028

**Published:** 2026-06-26

**Authors:** Elizabeth Randell, Claire Nollett, Vaso Totsika, Eilis Kennedy, Sean Johnson, Kim Smallman, Rachel McNamara, Jeremy Segrott, Jenifer Wakelyn, Angela Casbard, Kathy McKay, Ekaterina Bordea, David Wilkins

**Affiliations:** Centre for Trials Research, Cardiff University, UK; Division of Psychiatry, University College London, UK; Tavistock and Portman NHS Foundation Trust, London, UK; Comprehensive Clinical Trials Unit, University College London, UK; School of Social Sciences, Cardiff University, UK; Department of Clinical Educational and Health Psychology, University College London, UK; Institute of Population Health Sciences, University of Liverpool, UK

**Keywords:** Parent–child interaction, child development, communication, play, child-led

## Abstract

**Background:**

Mental health problems among children in England are rising, with significant wait times and barriers preventing many from accessing timely support. Watch Me Play! (WMP) is a caregiver–child interaction intervention designed to enhance child development and promote mental health resilience through child-led play.

**Aims:**

To assess the feasibility of delivering WMP remotely to parents and carers of children aged 0–8 years referred to UK early years and children’s services.

**Method:**

A non-randomised, single-group feasibility design with a mixed-methods process evaluation aimed to recruit 40 families. The study evaluated recruitment, retention, adherence, fidelity and acceptability. Outcomes were collected at baseline and 3 months; we conducted qualitative interviews to examine barriers and facilitators, and we used health economic data estimated intervention costs.

**Results:**

WMP was well-regarded and acceptable to families and service providers. Recruitment involved seven sites and 21 families, with 67% retention at 3 months. Self-reported adherence was 80%. Facilitators included the simplicity of the approach and quick access to support. Barriers included limited staff capacity and practitioner perceptions of readiness in families with complex needs. Hybrid delivery (online and face-to-face sessions) was feasible and acceptable. The average intervention cost was £209 per family.

**Conclusions:**

Findings indicate core feasibility parameters – including acceptability, fidelity, data-collection procedures and delivery across diverse contexts – were met. WMP is a low-cost intervention suited for early years services. Although a full-scale effectiveness trial is not yet warranted, a future randomised feasibility trial is recommended to investigate the acceptability of randomisation and recruitment across a broader range of services.

Around one in six children in England were identified as having a mental health problem in July 2020, up from one in eight in 2017.^1^ Early intervention is important to prevent or reduce the development of mental health problems.^2^ However, rising demand for services means families face long waits or cannot access support; approximately 75% of children facing a mental health problem experience delays that worsen their condition.^3^ Barriers are particularly pronounced for children with developmental delay and those living in areas of high deprivation^4,5^ despite elevated risk of developing mental health problems.^6^

Strengthening parent–child interaction and relationships is known to protect children’s mental health.^7^ Watch Me Play! (WMP) aims to enhance caregiver**–**child relationships and child development. WMP involves a parent observing their child at play and engaging in conversation about the play for up to 20 min per session. Some sessions are facilitated by a trained practitioner, in person or online, who provides prompts and guidance where necessary. WMP was first developed for children in care to promote mental health resilience through individual attention, age-appropriate stimulation and caregiver**–**child interaction. Evidence indicates that strengthening parent–child interaction can buffer against mental health difficulties and reduce symptom escalation.^2^ Since being manualised in 2018, it has been applied more widely across National Health Service (NHS) and third-sector child and family services with carers reporting improvements in relationships with their child, children’s play skills, language development and behaviour (J.W. 2016, 2018, unpublished; Koenig et al, unpublished; details of both available from J.W. (co-author of Koenig et al) on request via the corresponding author, E.R.). WMP shows promise as an early intervention for young children and, although not designed to replace specialist intervention, can be delivered early in the referral pathway providing timely support while families await specialist assessment. Before conducting a randomised controlled trial to demonstrate effectiveness, a feasibility study is needed to inform future trial design.

## Objectives and research questions

We aimed to assess the feasibility of delivering WMP to children aged 0–8 years in UK early years and children’s services, including recruitment and retention, adherence, intervention acceptability, implementation barriers and facilitators, and intervention costs. Early years and children’s services refers to NHS child development and psychology teams, specialist health visiting services, local authority family support teams and educational early years services working with children aged 0–8 years.

## Study design

We conducted a non-randomised, single-group feasibility study of the WMP intervention delivered remotely or in person for parents/carers of children aged 0–8 years who were referred to early years and children’s services. The study included a mixed-methods process evaluation examining recruitment, retention, fidelity, acceptability and contextual factors influencing implementation. Full methods are detailed by Randell et al in the peer-reviewed protocol article.^8^ A brief description follows.

## Method

### Study setting and participants

Sixteen services identified approximately 40 practitioners for training, but participation reduced to seven services because of staff turnover or delays with study set up. These were six NHS early years and children’s health services and one local authority education service. Practitioners included professionals such as clinical psychologists, assistant psychologists, social workers, specialist health visitors and child psychotherapists. Participants included parents/carers of eligible children (aged 0–8 years) referred to or accepted by these services, with consent obtained from the parents/carers.

The authors assert that all procedures contributing to this work comply with the ethical standards of the relevant national and institutional committees on human experimentation and with the Helsinki Declaration of 1975, as revised in 2013. All procedures involving human patients were approved by South Central – Berkshire B Research Ethics Committee (approval number 23/SC/0045). The study was registered on the International Standard Randomised Controlled Trial Number registry on 14 Apr 2023 (https://www.isrctn.com/ISRCTN13644899).

### Sample size

As this was a feasibility study, a formal sample size calculation was not conducted. The aim was to recruit up to 40 families (one caregiver per child) from a maximum of 15 sites over a 9-month period.

### Participant recruitment

Potentially eligible participants were identified by participating sites and provided with a brief information leaflet. From the leaflet, they were then signposted to the full participant information sheet and consent form. Those who provided informed consent (recorded as e-consent via Qualtrics) were screened for eligibility through an online questionnaire or a telephone interview with the research team. The online eligibility questionnaire included brief questions on child age, service involvement, developmental concerns, safeguarding status and the caregiver’s ability to engage in WMP sessions. Telephone screening covered the same areas, but allowed practitioners to clarify caregiving circumstances and any accessibility needs. Families were identified directly by practitioners working within early years and children’s services, typically based on professional judgement regarding suitability for a supportive, relationship-focused intervention during existing service involvement.

### Site training

Participating services nominated up to three practitioners each to undertake the study-specific WMP training. This was delivered in two 3 h online workshops, covering intervention principles, preparatory activities, case studies and available resources. A resource pack and a dedicated website provided additional tools, including an animated video for families.

### Intervention

**Fig. 1:**
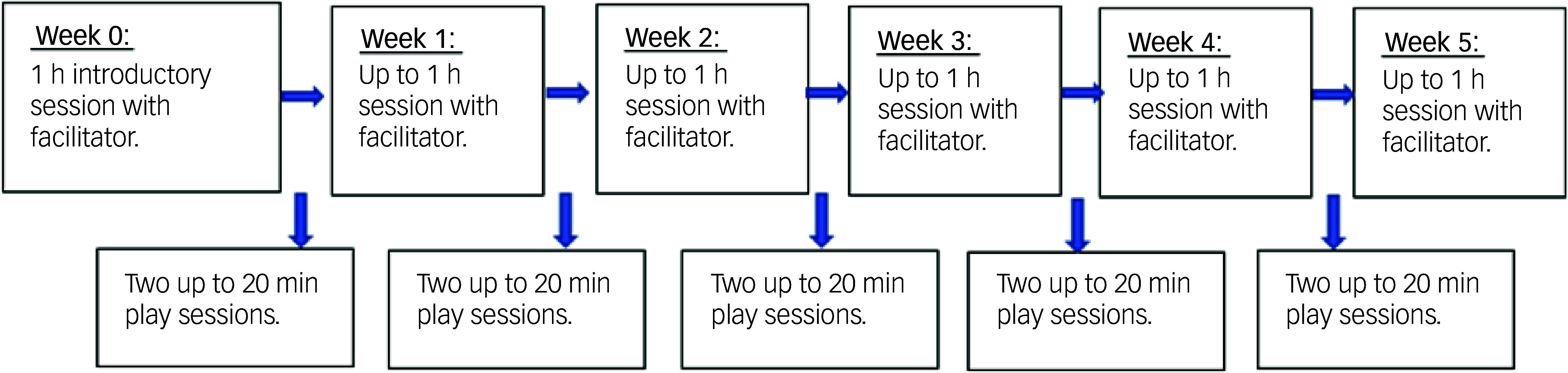
Fig. 1 long description. Watch Me Play! intervention flow diagram.

The planned intervention was intended to take place over a 5-week period (see Fig. 1). Services were asked to provide an introductory session to explain the intervention, followed by five facilitated sessions with parents. Additionally, parents were encouraged to complete ten independent sessions (two per week between facilitated sessions). This resulted in a schedule of three sessions per week, with one being facilitated. Adherence was defined as having done 10 out of 15 sessions, including the 5 facilitated ones.

Although WMP was intended to be primarily delivered online, practitioners offered face-to-face sessions when needed to support engagement thus creating a hybrid support model.^8^ Practitioners attended regular 75 min supervision meetings during the intervention period to discuss family interactions, play sessions and caregiver feedback. Practitioners also completed a short WMP checklist after each session with caregiver(s) (Supplementary File 1 available at https://doi.org/10.1192/bjo.2026.12028). This provided a framework for assessing key actions and fidelity related to facilitating the play sessions and covered stages such as preparation, supporting baby- or child-led play, watching the child play, and talking about the child’s play with both the child and the caregiver. Checklists were self-rated according to fidelity criteria to determine whether acceptable fidelity was achieved.

### Outcomes

The primary outcome was to determine the feasibility of delivering WMP. A mixed-methods approach was used to address study outcomes: (a) recruitment; (b) retention; (c) adherence to the intervention; (d) fidelity of WMP programme delivery; (e) assessment of the barriers and facilitators to implementation and variation across context (online or face to face); (f) acceptability of the study to parents, practitioners and service managers; (g) integration of WMP into existing service frameworks and (h) acceptability and feasibility of data collection procedures.

Along with feasibility outcomes, intervention mechanisms were explored through qualitative data gathered from interviews with parents/carers and delivery staff, focusing on their experiences with the WMP process and their perceptions of its impacts.

### Data collection

As part of evaluating the acceptability and feasibility of data collection procedures for a future trial, child/parent outcome and health economic measures (outlined in Table 1 below) were collected. Parents completed these measures via an online survey designed specifically for this study using Qualtrics (Qualtrics XM, 2023; Qualtrics International Inc., Seattle, Washington, USA; https://www.qualtrics.com), and through Q-Global and PARiConnect for the Vineland Adaptive Behavior Scale and Parent Stress Index, respectively. For participants who struggled with online data entry, participants were offered the option of telephone support from a researcher. We also collected data on safety. At baseline, the child’s developmental delay status was recorded via parent self-report, along with demographic details and information regarding the child’s contact with a social worker.

**Table 1:** Parent/carer-reported outcomes and health economic measures Table 1 long description.

Construct	Measure
Child mental health	Child Behavior Checklist^9^
	Strengths and Difficulties Questionnaire^10^
Child socialisation and communication	Vineland Adaptive Behavior Scale 3^11^
Parenting stress	Parent Stress Index – Short Form^12,13^
Parenting competence	Being a Parent Scale^14^
Parent–child relationship quality	Mothers’ Object Relations Scale – Short Form and Mothers’ Object Relations Scale (Child) for 0–4 years,^15,16^ Child–Parent Relationship Scale – Short Form for 5+ years,^17^ and the frequency of parent–child activities (Parent–Child Activity Index)^18^
Parent–child interaction	A 20 min videotaped free-play interaction between the parent/caregiver and the child (*n* = 8 baseline participants)
Parent/carer health-related quality of life and quality-adjusted life-years	EuroQol-5 Dimension, 5-Level^19^
Service use for child	A modified version of the Child and Adolescent Service Use Schedule^20^

Child and parent outcomes (Table 1) were collected at baseline and follow-up (3 months (±2 weeks) post-recruitment). Data on the child’s contact with a social worker (current and in the past), and reported developmental delay, were collected at baseline only. A subsample (*n* = 8) of participants also consented to record up to 20 min of play with their child via Microsoft Teams. The purpose of this was to assess the feasibility and acceptability of collecting data in this way in a future trial. A £50 voucher was offered for completion of each baseline and follow-up questionnaire. A £50 voucher was also offered to participants taking part in qualitative interviews, and those who took part in the free-play video recording.

Qualitative interviews were conducted virtually or by telephone with both participants and staff members delivering the intervention. Data collection was iterative, allowing preliminary analysis to guide the subsequent sampling decision and selection of further interviewees. We purposefully sampled interviewees with maximum variation across location. Coded identifiers were assigned to those taking part in interviews to maintain anonymity. Numerical codes were assigned to parents (e.g. 0103), and alphanumeric codes (e.g. P0101) were assigned to practitioners.

### Process evaluation

A mixed-methods process evaluation drew together quantitative and qualitative data.^21^ Quantitative methods were used to assess recruitment rates/patterns and intervention fidelity/adherence. Qualitative interviews with staff and managers (*n* = 13) and parents/carers (*n* = 4) examined implementation processes, intervention mechanisms and the role of contextual factors.

### Health economics

The health economic analysis aimed to estimate the cost of the WMP intervention and assess the feasibility of a full economic evaluation in a future effectiveness trial. We collected data on the number and duration of training sessions and staff’s time spent delivering the intervention to assess the cost of delivering facilitated WMP intervention. We assessed children’s healthcare service use via a modified version of the Child and Adolescent Service Use Schedule (CA-SUS) questionnaire. We assessed parent/carer health-related quality of life using the EuroQol-5 Dimension, 5-Level (EQ-5D-5L)^19^ and calculated quality-adjusted life-years. We costed the service use using national published sources.^22,23^ All costs were reported in 2021–2022 Great British Pounds.

### Progression criteria

No formal progression criteria were set for the study. It is hoped findings will help inform development of appropriate progression criteria for a subsequent randomised feasibility trial.

## Results

### Recruitment feasibility

**Fig. 2:**
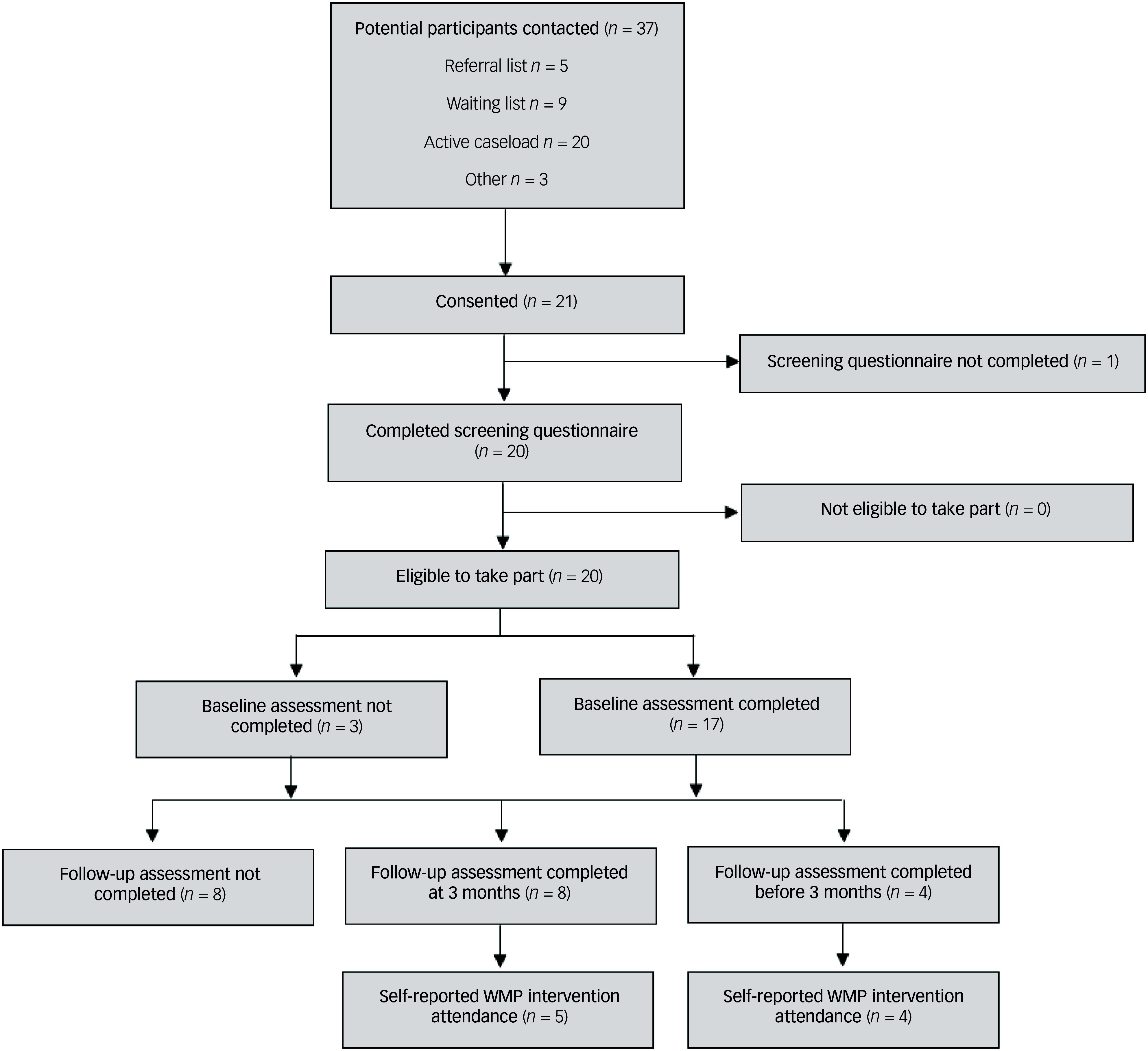
Fig. 2 long description. Flow diagram for the Watch Me Play! (WMP) study.

Delays in opening because of NHS ethics and governance approval processes meant that recruitment was reduced from 9 to 6 months. Recruitment was across seven sites, which opened at staggered times (subject to gaining approvals) over 3 months. Figure 2 shows the flow of participants through the study. Thirty-seven families were invited to participate, with 21 (57%) providing informed consent. Of these, 20 families completed the screening questionnaire, and all were eligible to take part. Baseline assessments were completed by 17 families (85%), and 12 families (60%) completed follow-up assessments. Among the nine families self-reporting WMP attendance, eight (89%) attended at least one session.

Of the 20 families who completed the screening questionnaire, 13 children (65%) had a reported neurodevelopmental disorder (either diagnosed or under assessment), including autism, global developmental delay or speech/language delay. Some children had more than one diagnosis. A full summary of conditions is detailed in Supplementary File 2, along with additional demographic details and developmental profiles. Seven families (35%) had contact with a social worker: two current, three within the past 24 months and two more than 24 months ago.

From qualitative interviews, parents reported that they found recruitment acceptable and appreciated clear explanations from practitioners:

‘But [named practitioner] explained everything […] and really reassured us that it was like, she explained that I don’t need to take part in it, if I don’t want to.’ (P0102)

They described feeling motivated to participate due to potential benefits for understanding their child and as a route for accessing more immediate help. One parent described ‘jumping’ at the opportunity having had no support or help for 2 years:

‘We had nothing else to um, I mean to go to, as a, as a help, as a support.’ (P0601)

The curtailed recruitment timeline increased staff workloads further constraining practitioner capacity, with many able to offer WMP to only one family at a time. Practitioners selected families they believed would benefit from WMP, prioritising those with existing relationships with the service over new referrals:

‘So, we were only approaching families who already had a care coordinator, within the service.’ (0103)

Practitioners mentioned that some families faced challenges related to technology access or anxiety about participating in online formats; however, because of the exploratory qualitative design, these issues were not quantified.

### Retention

Twenty families remained in the study until closure. Of these, 12 reached the 3-month time point and 8 (67%) completed at least 1 of the follow-up measures. Keeping recruitment open as long as possible meant that timelines for follow-up were reduced for some families. Subsequently, eight families were sent their follow-up questionnaires before reaching the 3-month time point. Four (50%) of these families completed at least one of the follow-up measures.

### Adherence to the intervention

Of the 12 families who reached the 3-month time point, 5 (42%) responded to the session questionnaire (Table 2). Four out of those five (80%) completed all five facilitated sessions and ten or more independent sessions. Out of 22 facilitated sessions in total, 11 were attended in person (50%) and 11 were attended online (50%). For the families who were sent the follow-up measures before reaching the 3 month time point (*n* = 8), four responded to the session questionnaire (50%). It must be noted that it is unlikely that these families had sufficient opportunity to complete all planned sessions.

**Table 2:** Session summary Table 2 long description.

Number of facilitated sessions attended	3-month follow-up	Pre-3-month follow-up
	*n* = 5	*n* = 4
Did not attend any facilitated sessions	0	1 (25%)
Attended at least one facilitated session	5 (100%)	3 (75%)
Attended one facilitated session	0	0
Attended two facilitated sessions	1 (2%)	0
Attended three facilitated sessions	0	2 (50%)
Attended four facilitated sessions	0	1 (25%)
Attended five facilitated sessions	4 (80%)	0
Number of attended facilitated sessions: median	5	3
Number of facilitated sessions attended online	11 (50%)	3 (30%)
Number of facilitated sessions attended face-to-face	11 (50%)	7 (70%)
Independent WMP sessions: median	11	4.5
Families who completed ten out of 15 sessions, including all five facilitated sessions	4 (80%)	0 (0%)

WMP, Watch Me Play! intervention.

### Fidelity

Fidelity checklists were self-rated by therapists: each of the five items was scored as achieved (2), partially achieved (1) or not yet achieved (0). A further question asked whether each item had been explored with the parent (1) or not (0). For a session to be completed with acceptable fidelity, it was expected that a score of 10 out of the maximum possible score of 15 was achieved.

Checklists for 68 sessions for 15 families were returned. Of those, 52 (76%) contained complete data for inclusion in the analysis. All 52 (100%) achieved a score of 10 or above. The median total fidelity score was 13 (out of 15). The remainder of the checklists were not included in the analysis as they were incomplete (*n* = 16, 24%). Practitioners described the checklist as a useful tool for reflecting on practice.

### Barriers and facilitators

Barriers and facilitators to implementing WMP were assessed through qualitative data from delivery staff and managers. This also included qualitative insights on how implementation varied across different service types.

#### Facilitators

##### Engagement and buy-in

Practitioners valued the intervention, with prior experience and training contributing to their willingness to participate. Supervision and professional development opportunities were also seen as beneficial.

##### Quick access to treatment and support

Parents appreciated the immediate access to support and were motivated to try something that could make a difference:

‘I thought, oh, it could be beneficial for, for my son, and you know, we could start right away, no waiting time, and it might you know, contribute to something bigger, you know, like, bigger study.’ (P0203)

##### Simplicity of the intervention

Delivery of WMP is simple, requiring no special facilities or equipment, making it accessible and well-received by both parents and practitioners:

‘For the parents it works with, it works really well, and it’s so simple, in its outlay and approach, that it’s a really good tool that anybody could use, with the right training.’ (0201)

#### Barriers

##### Evaluation of parent capacity and readiness

Practitioners described difficulties faced when trying to implement WMP where families had complex needs. They would often need to carefully introduce the intervention and support carers with readiness and confidence to participate fully:

‘I think if … if you’ve got a very vulnerable adult or carer, who is … whose needs or understanding are … they need their needs met perhaps more or alongside this, because this is very much completely child focused, it’s child led, and … and … and for some parents/carers, to have that confidence to just let the child get on with something, I think would be very … would be quite a challenge.’ (1501)

##### Staffing capacity

Practitioners highlighted unexpected demands on their time, including additional administrative tasks and the need to balance WMP with regular caseloads, posed challenges:

‘I’m probably gonna offer a little bit to this boy with his dad, because it, it wouldn’t have really been helpful, to put all of them together. So, it ends up being a bit more work, I guess than what I expected at the beginning.’ (0202)

##### Fit with services

The intervention’s integration within existing service frameworks was complex, particularly for families with high needs. Further work is needed to understand how WMP fits across services with diverse care pathways.

Delays in recruitment and the limited time frame for delivering the intervention further strained resources and impacted service delivery. Suggestions for improvement from sites included better clarification of roles and what to expect over the course of the study, to manage caseloads:

‘... there was some, um, challenges in identifying families because of, just because of the process of research, as it is, it’s kind of, when, when we would need them for, you know, the deadlines, kind of got shifted and things like that. So, it was, we weren’t know, we didn’t know exactly when we would need to start the piece of work.’ (0202)

### Acceptability of the study to parents, practitioners and service managers

Parents and staff described a positive experience and good level of acceptability in taking part in the study:

‘Um, no, everybody was really kind, and quick to respond, um, yeah, it was a fantastic study, everybody was really dedicated, um and very kind, so, yeah, I’m really, really happy that I participated, yeah.’ (P0203)

Parents stated that information provided was clear, helping them understand what the study involved. Practitioners found the intervention valuable and appreciated the training and supervision offered:

‘I was very interested in the training, for Watch Me Play!, it’s a good fit with the clients that we work with.’ (0201)

Although some parents appreciated the flexibility of online delivery, especially in terms of scheduling and comfort, there were instances where parents felt this wasn’t appropriate in their circumstances and preferred face-to-face delivery. Some practitioners also noted concerns around the challenges of working online, particularly with children with autism or high needs:

‘Um, if it’s gonna be a face-to-face thing, then we’re definitely going to do it, because it’s a different thing, with an autistic child, you can’t really do online things, he’s not gonna pay attention for 2 or 3 minutes and that’s more than…he can do, I mean his attention span is quite short. And, keeping him online, in front of a computer, is absolutely non-, non-viable.’ (P0601)

Practitioners were open to online delivery of WMP for certain families and situations, but appreciated the option of face-to-face delivery for its ease of engagement and ability to observe interactions closely. Overall, practitioners preferred face-to-face delivery, given this was where most of their experience lay. Other factors that influenced delivering WMP online included technological capabilities, practical considerations, family needs and the effectiveness of therapeutic connections:

‘And, then we had a go online, for, you know, to start online, but there, there was lots of sort of problems with technology, she kind of, couldn’t manage to get into the link, and there was a, it, kind of, lots of things, made it quite disrupted.’ (0202)

Some practitioners described limitations in being able to clearly observe the child/parent interactions, but in practice, this resulted in asking the parent to narrate the play more:

‘When we were online, and then, mum became more active in that sense…cos I actually had to say, you know…I’m not quite sure what the little girl was saying…I wonder if you can tell me? You know…and then mum took more of an active role…so, in some way, it paradoxically, also helped.’ (0101)

Although practitioners highlighted their concerns, reservations and challenges with online delivery, successes were also reported. There was an openness from practitioners to delivering WMP online and appreciation for researching this mode of delivery:

‘I think it worked … I was surprised by how well it worked, and I would guess that it depends on the families that are able to use it as well…’ (0201)

Practitioners and site leads reported challenges with the volume of paperwork, complex study processes and delays in securing approvals, which were especially overwhelming for those new to research on this scale. However, communication with the research team was praised, with participants feeling supported throughout. Despite some logistical and technological barriers, the intervention and research process were viewed positively, with the flexibility of online delivery and the support from the research team being key factors.

### Integration of WMP into existing service pathways

WMP was generally offered when families were waiting for other support, although it was not always seen as a first-line intervention. Some practitioners carefully considered whether it was appropriate for families based on their specific needs and stage in the service process. In many cases, WMP was offered alongside other services, rather than as a stand-alone or ‘holding’ intervention. It was integrated into ongoing relationships with families, ensuring continuity in care. Once WMP was offered, practitioners often continued their involvement with families beyond the study period.

### Acceptability and feasibility of data collection procedures

Parents reported positive experiences of taking part in WMP with varying preference for accessing questionnaires via phone or laptop. Completion rates of the measures were relatively low (Table 3) across both baseline and follow-up time points. Because the purpose of collecting Table 3 measures was to evaluate acceptability and feasibility rather than clinical change, questionnaire scoring and outcome analysis were not conducted. Because of the small number of follow-up respondents and incomplete data across measures, deriving and reporting scores would not have yielded meaningful or generalisable results. Instead, findings focus on the feasibility issues parents raised to inform measure selection in a future feasibility randomised controlled trial.

**Table 3:** Outcome measure completion rates Table 3 long description.

Outcome measure	Baseline, *n* (%)	3-month follow-up *n* (%)
Parenting competence		
Being a Parent Scale	15/20 (75)	12/20 (60)
Parent–child relationship		
Mothers’ Object Relations Scale – Short Form 0–4 years	12/15 (80)	10/15 (66.6)
Child–Parent Relationship Scale 4+ years	2/5 (40)	1/5 (20)
Child–Parent Activity Index	13/20 (65)	11/20 (55)
Emotional Availability Scales	8/8 (100)	
Child mental health		
Child Behavior Checklist 1.5–5 years	11/16 (68.75)	11/16 (68.75)
Child Behavior Checklist 6–18 years	0/1 (0)	0/1 (0)
Strengths and Difficulties Questionnaire 2–17 years	11/17 (64.7)	11/17 (64.7)
Parent/carer health-related quality of life and quality-adjusted life-years		
EuroQol-5 Dimension, 5-Level	13/20 (65)	12/20 (60)
Service use for child		
Modified version of the of the Child and Adolescent Service Use Schedule	12/20 (60)	12/20 (60)
Parenting stress		
Parent Stress Index – Short Form	10/20 (50)	11/20 (55)
Child socialisation and communication		
Vineland Adaptive Behavior Scale 3	7/16 (43.75)	7/16 (43.75)

Some parents required significant support to complete the questionnaires, which was manageable with the small sample size, but could be challenging in a larger study. Practitioners reported challenges with the screening and consent process, although parents did not report any significant issues. The forms were off-putting for some families, particularly those with fewer resources or limited English proficiency, which led to additional support needed from practitioners.

One measure was particularly challenging with ten parents expressing concerns about the Vineland measure,^11^ especially its relevance for younger children (3–5 years), as many of the questions were not age-appropriate. Some parents suggested that the questionnaire could be filtered based on age, and others noted that their children were non-verbal or too young for the questions asked.

Parents had initial concerns about the logistics of video recording free play and the child staying within the frame. The study achieved the recruitment target of eight videos, but only 64% were able to be coded. Practical challenges emerged leading to data loss, such as limited camera width from mobile phones, the child and/or the parent being off screen and the conversation taking place in a language that the researcher could not code. These factors affected the quality and length of the video data, with three of the eight videos not codable at all and only one of the eight videos achieving the 20 min duration target. In addition, for the age range targeted in this study (0–8 years), there is only one suitable observational coding scheme (Emotional Availability Scales, version 4.1),^24^ restricting choice for observational analyses. Overall, the feasibility of using remote video recording of free play as an additional, objective evaluation of parent–child interaction and attunement was low.

### Intervention mechanisms

Both parents and practitioners reported initial doubts or nervousness about the intervention, particularly regarding being observed and understanding the model. As sessions progressed, these concerns diminished, and positive outcomes were reported. Practitioners emphasised the importance of explaining the model clearly, and noted that parents often needed several sessions to fully grasp that WMP was for both the parent and the child, focusing on shared observation and engagement:

‘Yeah, it was, the first session I was a little bit nervous, cos I didn’t really know what to expect, obviously, it’d all been explained to us. Um, but you don’t know til you get in there kind of, thing, um, but it was honestly, nothing to be worried about, it was absolutely fine.’ (P0102)

Parents valued the one-on-one, intentional time spent with their child, noting it allowed for shared enjoyment and deepened their connection. The intervention encouraged child-led play, requiring parents to shift from a teaching role to a more child-focused observing role. This was challenging for some, but became more natural over time as they understood the value of letting the child lead the interaction.

Parents and practitioners reported improved parent/child relationships, marked by greater engagement and less frustration. These themes were consistent across interviews with both parents and practitioners. There were reported improvements in communication, with some children becoming more expressive and able to communicate their wants and needs. Parents also described feeling less stressed and more present with their children, leading to more positive interactions:

‘So, the whole process, has been quite a big one for us, unlearning what we’d learnt before, and learning to be more free, and just playful with him.’ (P0602)

Parents reported extending the principles of WMP into daily routines with all children in the family:

‘While eating, while we’re playing, it’s not about just Watch Me Play!, it’s about watch me and I will watch you guys back [chuckling].’ (P0602)

Ending the relationship abruptly after the intervention was viewed as potentially harmful, leading to ongoing support needs for both families and practitioners:

‘And I think any interventional programme, […] if it’s going to have the impact you want on it, it’s … you have to be mindful that if you have been working with those families, weekly, and then you just stop, that can sometimes have worse effects than never even starting in the first place.’ (1501)

Parents expressed how having a deeper understanding of their child’s needs, helped them communicate more effectively with other professionals, allowing them to advocate better for their child’s support.

### Safety

In addition to the standard International Council for Harmonisation – Good Clinical Practice serious adverse event reporting requirements, for the purposes of this study, the removal of a child from the biological family (or unplanned removal more specifically) was considered to be an adverse event. No adverse events were reported.

### Health economics

Sixteen practitioners participated, with training costing £7986 (£499.1 per practitioner). Practitioners attended an average of five out of four online supervision meetings, costing £665 per practitioner. The total cost of delivering the intervention for 20 children attending all 6 sessions was £8359 (£418 per child). With an average of three sessions attended, the cost per child was £209, with a range of £209 to £418 depending on attendance and delivery mode. Direct comparison with treatment as usual was not possible as an estimate of these was not included in this work.

The feasibility of collecting service use data and health-related quality-of-life information for future cost-effectiveness analysis was promising, 60% of participants had complete data on health-related quality of life and 50% for the CA-SUS questionnaire. Parents’/carers’ EQ-5D-5L scores were 0.85 (s.d. 0.22) on average at baseline and 0.89 (s.d. 0.09) on average. Mean cost of healthcare service use per participant was £346 (s.d. 432) at baseline and £859 (s.d. 906) at 3-month follow-up. Low response rates will be a potential challenge for future evaluations.

## Discussion

This study marks the first evaluation of the feasibility of delivering WMP across a range of early years and family services in England. Specifically, the feasibility of recruitment, retention, acceptability, fidelity and data collection processes, rather than to the clinical effectiveness of WMP. Despite recruitment challenges, the study achieved significant success in engaging families with social care involvement (35%) and children with developmental disabilities (65%). The recruited sample demonstrated good diversity in terms of child ethnicity (50% from a Black, Asian or minority ethnic background), parent disability (35%) and parent educational level (55% below university degree).

Retention rates were good at 67%, and self-reported adherence was high at 80%. However, only 42% of recruited participants reported on their session completion, meaning conclusions on adherence feasibility for future studies cannot be firmly established.

Despite significant initial interest from services and training approximately 40 staff members, only 16 (40%) ultimately delivered WMP to families because of delays in study initiation and staff turnover. Nevertheless, strong buy-in for WMP emerged as a crucial facilitator for the recruitment of sites and staff. The good fit of WMP with family needs, the accessible delivery format and its compatibility with existing treatment as usual support its potential within these diverse services. Reported barriers primarily concerned (staff perception of) parent readiness and staff capacity to deliver a new intervention alongside existing workloads, a common challenge in health service evaluations. The high level of staff and service buy-in underscores the need for further investigation into WMP’s potential in early years and family services. Future evaluations should carefully consider the timing of training and delivery to ensure closer proximity and explore strategies to increase training and supervision capacity to accommodate a larger number of practitioners.

Although WMP was originally intended primarily for online delivery, practitioners provided in-person sessions when required to enhance participant engagement, leading to a hybrid model that was feasible and acceptable. Parents appreciated the accessibility of remote sessions, whereas staff often preferred in-person delivery for building rapport. The flexible delivery format was particularly beneficial for families of children with neurodevelopmental conditions, allowing for customisation to suit individual family and child needs. This aligns with the importance of flexibility and personalisation in service provision for families with complex needs. The existing use of a hybrid format for WMP, initially necessitated by COVID-19 restrictions, makes it particularly suitable for reaching diverse populations. This too fits with the current NHS focus on technological advances and innovation in health and care.

Staff preferred offering WMP to families they already knew, limiting its use for waitlisted or referred families. This has implications for WMP training and how staff are supported to integrate the intervention into their existing services. WMP can serve as a first-line intervention, helping engagement with families,^25^ which could be of great value to families on referral and waiting lists. Waiting list interventions show promise and are increasingly being taken up by child mental health services (especially interventions addressing parents/caregivers) in an effort to address long waiting lists.^26^

Recruiting across this wider range of sites, particularly non-NHS, requires further investigation. The next step is a feasibility randomised controlled trial across NHS, social care, integrated early years and family services, schools, education settings and third-sector organisations. This direction is supported by the interest in WMP from the not-for-profit family support movement, Home-Start^27^ and ongoing staff training.

This initial evaluation indicates that WMP is well-regarded and seen as valuable by both service providers and families. Insights gained regarding recruitment challenges, participant engagement and data collection methods will be crucial in informing the design of future evaluations. This includes adapting WMP training to offer it earlier in the service pathway and modifying research evaluation methods to better align with the needs of parents and the supporting delivery sites. The feasibility of a health economics evaluation was supported, and the preliminary costing of WMP shows promise, with an average cost of £209 per family, ranging up to £494 for a per-protocol face-to-face implementation. This compares favourably with cost estimates for similar interventions.^28^ The proposed hybrid delivery model was well-received, with initial indications of improved staff acceptability. Remaining questions regarding the acceptability of randomisation and recruitment feasibility across a wider range of services necessitate a feasibility randomised controlled trial as the next step.

### Limitations

Challenges with recruitment, staff turnover and delays in study initiation limited the number of sites and participants. Although the hybrid model was well-received, it remains unclear how well it can be scaled across different service types, particularly non-NHS settings. Voucher compensation may have influenced participant engagement, potentially inflating adherence to research procedures. Although common in feasibility studies, this incentive may not reflect engagement levels in routine practice. Participant identification relied on practitioner judgement, which may have resulted in recruiting families perceived as more ready or easier to engage, limiting generalisability. Further investigation into recruitment and randomisation feasibility is needed, especially in diverse service settings.

## Supporting information

10.1192/bjo.2026.12028.sm001 Randell et al. supplementary material 1 Randell et al. supplementary material

10.1192/bjo.2026.12028.sm002 Randell et al. supplementary material 2 Randell et al. supplementary material

## Data Availability

The data that support the findings of this study are available from the corresponding author, E.R., upon reasonable request.

## Supplementary material

The supplementary material is available online at https://doi.org/10.1192/bjo.2026.12028

## Fig. 1 Long description

The flowchart outlines the Watch Me Play intervention process over five weeks. It begins with a one-hour introductory session with a facilitator in Week 0, followed by two play sessions of up to 20 minutes each. Weeks 1 through 5 each include up to one hour of session with the facilitator and two play sessions of up to 20 minutes each. The flowchart visually represents the structured approach to early intervention for mental health support in children.

Navigate back to Fig. 1.

## Table 1 Long description

The table presents constructs and their corresponding measures for parent/carer-reported outcomes and health economic measures. It includes eight rows and two columns. The first column lists constructs such as child mental health, child socialisation and communication, parenting stress, parenting competence, parent-child relationship quality, parent-child interaction, parent/carer health-related quality of life and quality-adjusted life-years, and service use for child. The second column lists the measures associated with each construct, including Child Behavior Checklist, Strengths and Difficulties Questionnaire, Vineland Adaptive Behavior Scale 3, Parent Stress Index - Short Form, Being a Parent Scale, Mothers’ Object Relations Scale - Short Form and Mothers’ Object Relations Scale (Child) for 0-4 years, Child-Parent Relationship Scale - Short Form for 5+ years, and the frequency of parent-child activities (Parent-Child Activity Index), a 20-minute videotaped free-play interaction, EuroQol-5 Dimension, 5-Level, and a modified version of the Child and Adolescent Service Use Schedule.

Navigate back to Table 1.

## Fig. 2 Long description

Flow diagram for the Watch Me Play! (WMP) study. The diagram begins with 37 potential participants contacted, divided into referral list (5), waiting list (9), active caseload (20), and other (3). 21 participants consented, with 1 not completing the screening questionnaire. 20 completed the screening questionnaire, all found eligible. 3 did not complete the baseline assessment, while 17 did. Of those who completed the baseline assessment, 8 did not complete the follow-up assessment at 3 months, while 8 did. 4 completed the follow-up assessment before 3 months. 5 reported WMP intervention attendance after 3 months, and 4 reported it before 3 months.

Navigate back to Fig. 2.

## Table 2 Long description

The table compares session attendance between two groups: 3 month follow-up with five participants and pre-3 month follow-up with four participants. It details attendance at facilitated sessions, including the number of sessions attended, median sessions, and online versus face-to-face attendance. For the 3 month follow-up group, all participants attended at least one session, with four attending all five sessions and a median of five sessions. Half of the sessions were attended online and half in person. In the pre-3 month follow-up group, three attended at least one session, with two attending three sessions and a median of three sessions. Seven percent of sessions were attended in person and thirty percent online. Additionally, the table notes that eighty percent of the 3 month follow-up group completed ten out of fifteen sessions, including all five facilitated sessions, while none in the pre-3 month follow-up group did so.

Navigate back to Table 2.

## Table 3 Long description

The table presents the completion rates of various outcome measures at baseline and 3-month follow-up. It includes data on parenting competence, parent-child relationship, child mental health, parent/carer health-related quality of life, service use for child, parenting stress, and child socialization and communication. The table has 16 rows and 3 columns, with column headers being Baseline, n (percentage) and 3 month follow-up n (percentage). Notable measures include Being a Parent Scale, Mothers’ Object Relations Scale, Child Behavior Checklist, EuroQol-5 Dimension, Parent Stress Index, and Vineland Adaptive Behavior Scale. The data shows varying completion rates across different measures and time points, with some measures having higher completion rates at baseline compared to follow-up.

Navigate back to Table 3.
