# Supplementary material for: Watch Me Play!: results of a feasibility study of a remotely delivered intervention to promote mental health resilience for children (age 0–8 years) across UK early years and children’s services
Source: BJPsych Open. 2026 Jun 26;12(4):e171. doi: 10.1192/bjo.2026.12028 (PMC13312274; doi:10.1192/bjo.2026.12028)
Supplement: Randell et al. supplementary material 1 — Randell et al. supplementary material [file S2056472426120286sup001.docx]

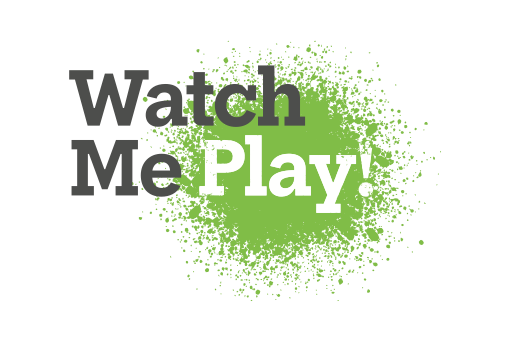


**WMP checklist for practitioners**

Practitioner name: …

Session date: …

Parent and child initials: …

Age of child: …

**1. Preparation:**

Guiding parents or carers to select up to 6 age-appropriate toys; asking parents or carers to put away electronic and battery toys, screens, phones away and turn TV off; sitting with parents or carers on or near the floor; encouraging parents or carers to help the child to prepare for the end of the play session.

Achieved **2**  Partially achieved **1** Not yet achieved **0**

Explored with caregiver? Yes **1** No **0**

**2. Baby or child-led play:**

Supporting the parent or carer to allow the baby or child to take the lead and play freely, and to join in if invited to by the child, but still following the child’s lead; encouraging and guiding the parent to avoid teaching, correcting, directing or tidying up during the WMP time.

Achieved **2**  Partially achieved **1** Not yet achieved **0**

Explored with caregiver? Yes **1** No **0**

**3. Watching the baby or child play:**

Supporting the parent or carer to give their undivided attention to whatever their baby or child chooses to do, encouraging the parent or carer to watch their baby or child and see how they respond, giving the baby or child time to respond and find their own way in play

Achieved **2**  Partially achieved **1** Not yet achieved **0**

Explored with caregiver? Yes **1** No **0**

**4. Talking with the baby or child about their play:**

Describing what the baby or child does and encouraging the parent or carer to do the same; with a baby, echoing their sounds and vocalisations and encouraging the parent or carer to do the same; guiding and encouraging the parent to talk with their baby or child about their play using simple language or sounds

Achieved **2**  Partially achieved **1** Not yet achieved **0**

Explored with caregiver? Yes **1** No **0**

**5. Talking with another adult about the child’s play:**

Talking with the parent or carer the child’s play in the last play session - what they noticed, any changes or lack of change, and reflecting on how it feels to be with their child while they are playing; sharing their own observations about moments of connectedness, developments and difficulties, linking with the parent or carer’s goals, if these have been agreed; problem-solving with parents or carers about what is difficult for them in WMP and what could help

Achieved **2**  Partially achieved **1** Not yet achieved **0**

Explored with caregiver? Yes **1** No **0**

**Was this session online or in-person?**

Date case last discussed in supervision/work discussion:

Comments /observations:

Fidelity Score for this session:

If the Fidelity Score is less than 10, please re-read the WMP Short Guide, Manual for Parents and Further Information.

**WMP checklist for practitioners V3 1/6/2023 ©Jenifer Wakelyn 2023**
