# Supplementary material for: Watch Me Play!: results of a feasibility study of a remotely delivered intervention to promote mental health resilience for children (age 0–8 years) across UK early years and children’s services
Source: BJPsych Open. 2026 Jun 26;12(4):e171. doi: 10.1192/bjo.2026.12028 (PMC13312274; doi:10.1192/bjo.2026.12028)
Supplement: Randell et al. supplementary material 2 — Randell et al. supplementary material [file S2056472426120286sup002.docx]

**Supplementary File 2:** Baseline characteristics

|  | n (%) |
| --- | --- |
| Age: (years) median [IQR] | 34.5 [32.0;39.5] |
| Gender: |  |
| Male | 2 (10%) |
| Female | 18 (90%) |
| Another gender | 0 (0%) |
| Prefer not to say | 0 (0%) |
| Missing | 0 (%) |
| Ethnicity: |  |
| Asian/Asian British: Indian | 0 (0%) |
| Asian/Asian British: Pakistani | 0 (0%) |
| Asian/Asian British: Bangladeshi | 2 (10%) |
| Asian/Asian British: Chinese | 0 (0%) |
| Asian other (please describe) | 1 (5%) |
| Black/African/Black British: African | 0 (0%) |
| Black/African/Black British: Caribbean | 0 (0%) |
| Black other (please describe) | 0 (0%) |
| Mixed/multiple ethnic groups: White and Black Caribbean | 0 (0%) |
| Mixed/multiple ethnic groups: White and Black African | 0 (0%) |
| Mixed/multiple ethnic groups: White and Asian | 0 (0%) |
| Mixed other (please describe) | 0 (0%) |
| Other Ethnic group: Arab | 0 (0%) |
| Ethnic other (please describe) | 0 (0%) |
| White: English/Welsh/Scottish/Northern Irish/British | 11 (55%) |
| White: Irish | 2 (10%) |
| White: Travelling community | 0 (0%) |
| White: Other (Please describe) | 0 (0%) |
| Any other ethnic background (Please describe) | 0 (0%) |
| Prefer not to say | 3 (15%) |
| Missing | 1 (5%) |
| How is your health in general? |  |
| Very good | 6 (30%) |
| Good | 9 (45%) |
| Fair | 5 (25%) |
| Bad | 0 (0%) |
| Very bad | 0 (0%) |
| Missing | 0 (0%) |
| Do you have a longstanding illness, disability or infirmity? |  |
| Yes | 7 (35%) |
| No | 13 (65%) |
| Missing | 0 (0%) |
| Highest level of educational qualifications: |  |
| No qualifications | 2 (10%) |
| Some GCSEs passes or equivalent | 4 (20%) |
| 5 or more GCSEs at A*-C or equivalent | 0 (0%) |
| 5 A/AS Levels or equivalent | 2 (10%) |
| Higher Education but below degree level | 3 (15%) |
| Degree (e.g. BA, BSC, MA) | 9 (45%) |
| Don’t know | 0 (0%) |
| Prefer not to say | 0 (0%) |
| Missing | 0 (0%) |
| Occupation: |  |
| Employed full time (30+ hours/week) | 7 (35%) |
| Employed part-time (or variable hours) | 3 (15%) |
| Employed but on maternity/paternity leave, sick leave | 1 (5%) |
| Full time parent/carer | 7 (35%) |
| Not working and looking for work | 1 (5%) |
| Not working and not looking for work | 1 (5%) |
| Missing | 0 (0%) |
| Do you feel that you will be able to complete questionnaires in English? |  |
| Yes | 20 (100%) |
| No | 0 (0%) |
| Missing | 0 (0%) |
| Are you already doing Watch Me Play outside of this study? |  |
| Yes | 0 (0%) |
| No | 20 (100%) |
| Missing | 0 (0%) |
| Relationship to the child: |  |
| Biological mother | 16 (80%) |
| Biological father | 1 (5%) |
| Adoptive mother | 0 (0%) |
| Adoptive father | 1 (5%) |
| Stepmother | 0 (0%) |
| Stepfather | 0 (0%) |
| Foster mother | 1 (5%) |
| Foster father | 0 (0%) |
| Grandmother | 0 (0%) |
| Grandfather | 0 (0%) |
| Other | 1 (5%) |
| Missing | 0 (0%) |
| Child's current age: |  |
| Below 18 months | 3 (15%) |
| 18 months to 23 months | 0 (0%) |
| 2 years old | 1 (5%) |
| 3 years old | 8 (40%) |
| 4 years old | 3 (15%) |
| 5 years old | 4 (20%) |
| 6 years old | 1 (5%) |
| 7 years old | 0 (0%) |
| 8 years old | 0 (0%) |
| Older than 8 years old | 0 (0%) |
| Missing | 0 (0%) |
| Child's gender: |  |
| Male | 12 (60%) |
| Female | 8 (40%) |
| Another gender | 0 (0%) |
| Prefer not to say | 0 (0%) |
| Missing | 0 (0%) |
| Does your child have a longstanding illness, disability or infirmity? |  |
| Yes | 5 (25%) |
| No | 15 (75%) |
| Missing | 0 (0%) |
| Children with reported neurodevelopmental condition: |  |
| Yes | 13 (65%) |
| No | 7 (35%) |
| Missing | 0 (0%) |
| (A breakdown of reported neurodevelopmental conditions)  Conditions professionals have diagnosed or have told you might apply to your child: |  |
| Leaning disability or intellectual disability (diagnosed) | 2 (10%) |
| Leaning disability or intellectual disability (under assessment) | 6 (30%) |
| Developmental Delay or Global Developmental Delay (diagnosed) | 1 (5%) |
| Developmental Delay or Global Developmental Delay (under assessment) | 5 (25%) |
| Autism or Autistic Spectrum Disorder (diagnosed) | 4 (20%) |
| Autism or Autistic Spectrum Disorder (under assessment) | 7 (35%) |
| Down Syndrome (diagnosed) | 0 (%) |
| Down Syndrome (under assessment) | 0 (%) |
| ADHD (diagnosed) | 0 (0%) |
| ADHD (under assessment) | 2 (10%) |
| Motor Disorders (diagnosed) | 0 (0%) |
| Motor Disorders (under assessment) | 1 (5%) |
| Cerebral palsy (diagnosed) | 0 (0%) |
| Cerebral palsy (under assessment) | 1 (5%) |
| Specific Learning Difficulty (diagnosed) | 0 (0%) |
| Specific Learning Difficulty (under assessment) | 1 (5%) |
| Communication disorder (diagnosed) | 0 (0%) |
| Communication disorder (under assessment) | 2 (10%) |
| Foetal Alcohol Syndrome (FASD) (diagnosed) | 0 (0%) |
| Foetal Alcohol Syndrome (FASD) (under assessment) | 0 (0%) |
| A genetic neurodevelopmental syndrome (diagnosed) | 0 (0%) |
| A genetic neurodevelopmental syndrome (under assessment) | 1 (5%) |
| A mental health problem (diagnosed) | 0 (0%) |
| A mental health problem (under assessment) | 2 (10%) |
| Are you or have you ever been in contact with social services because of your child? |  |
| Yes, currently in contact with social services because of my child | 2 (10%) |
| Yes, previously in contact with social services because of my child within the past 24 months | 3 (15%) |
| Yes, previously in contact with social services because of my child but not within the past 24 months | 2 (10%) |
| No, never been in contact with social services because of my child | 13 (65%) |
| Missing | 0 (0%) |
